# Recombinant human endostatin combined with radiotherapy inhibits colorectal cancer growth

**DOI:** 10.1186/s12885-017-3903-3

**Published:** 2017-12-28

**Authors:** Ke Zhang, Ye Wang, Xiaoli Yu, Yanyan Shi, Yasai Yao, Xiaofang Wei, Xuezhen Ma

**Affiliations:** 1 Graduate School, Qingdao University, Qingdao, Shandong 266071 People’s Republic of China; 2 Department of Oncology, Hospital of Qingdao Commercial Staff, Qingdao, Shandong 266011 People’s Republic of China; 3 Clinical Laboratory, Qingdao Central Hospital, The Second Affiliated Hospital of Medical College of Qingdao University, Qingdao, Shandong 266042 People’s Republic of China; 4 Department of Oncology, Qingdao Central Hospital, The Second Affiliated Hospital of Medical College of Qingdao University, No. 127 Siliunan Road, Qingdao, Shandong 266042 People’s Republic of China

**Keywords:** Recombinant human endostatin, Radiotherapy, Colorectal cancer HCT-116 cells, Antitumor, Angiogenesis

## Abstract

**Background:**

To examine the effects of recombinant human endostatin combined with radiotherapy on colorectal cancer HCT-116 cell xenografts in nude mice.

**Methods:**

Forty male BALB/c nude mice were injected with human colorectal cancer HCT-116 cells to form xenografts and then randomized into the following 4 groups (each group comprised ten mice): a control group, an endostatin group (20 mg/kg endostatin once a day for 10 days), a radiotherapy group (a 6-Gy dose was administered via a 6-MV X-ray on day 5 post-inoculation), and a combination therapy group (radiotherapy with endostatin treatment). The tumor growth inhibition rate were detected. CD31, vascular endothelial growth factor (VEGF), and hypoxia inducible factor-1α (HIF-1α) expression and microvascular density (MVD) were evaluated by immunohistochemistry. The expression of VEGF protein was also detected by western blotting.

**Results:**

The tumor growth inhibition rate in the radiotherapy with endostatin treatment group was significantly higher than those in endostatin group or radiotherapy group (77.67% vs 12.31% and 38.59%; *n* = 8 per group, *P* < 0.05). The results of immunohistochemistry showed that treatment with radiotherapy induced significant increases in CD31, VEGF, and HIF-1α expression and MVD compared with treatment with saline, while treatment with endostatin or radiotherapy with endostatin induced reductions in CD31, VEGF, and HIF-1α expression and MVD compared with treatment with saline (*n* = 8 per group, *P* < 0.05). The results of western blotting showed that VEGF protein expression in radiotherapy group was significantly increased compared with that in the control group. However, VEGF protein expression in the endostatin or radiotherapy with endostatin groups was significantly decreased compared with that in the control group (*n* = 8 per group, *P* < 0.05).

**Conclusions:**

Endostatin combined with radiotherapy can significantly inhibit HCT-116 cell xenograft growth, possibly by inhibiting angiogenesis and attenuating tumor cell hypoxia.

## Background

Colorectal cancer is the most common malignant tumor of the digestive system [1]. Dietary changes have resulted in significant increases in the incidence of and mortality associated with colorectal cancer in China in recent years [2], and the age of colorectal cancer onset in China has decreased significantly compared with that in European and American countries (mean age: 45 years). Rectal cancer accounts for 60%–75% of colorectal cancer cases in China and for 45% of colorectal cancer cases in European and American countries [3]. Most patients with colorectal cancer have locally advanced disease at the time of their diagnosis and treatment [4]. However, the overall colorectal cancer survival rate remains poor despite advances in colorectal cancer treatment. Radiotherapy is an important treatment for locally advanced rectal cancer, and many patients respond poorly to RT because of tumor cell hypoxia [5, 6].

Hypoxia is one of the basic characteristics of the solid tumor microenvironment. In tumor cells, the G2/M phase shortens significantly in the setting of hypoxia. Tumor cells in the G2/M phase are more sensitive to radiation than cells in other phases of the cell cycle; thus, hypoxia induces resistance to radiation in tumor cells by shortening the G2/M phase [7].

Folkman first proposed the tumor angiogenesis theory in the New England Journal of Medicine in 1971, the year in which he also demonstrated that antiangiogenesis agents can inhibit tumor growth [8]. In recent decades, a variety of antiangiogenic drugs targeting tumor endothelial cells have been used in combination with radiotherapy and chemotherapy to improve the cure rates of patients with cancer and to prolong survival times in such patients. Antiangiogenesis is an important target in tumor biological therapy, and the idea that antiangiogenic drugs can attenuate tumor cell hypoxia and thus enhance radiosensitivity has become a popular topic in the field in recent years [9].

Recombinant human endostatin (Endostar) is one of the most potent angiogenesis inhibitors of angiogenesis developed independently in China. Endostatin can significantly inhibit the proliferation and migration of vascular endothelial cells and the formation of new blood vessels to prevent tumor cells from receiving the nutrients necessary for growth and metastasis [10]. Some studies have also shown that endostatin can inhibit lymph node metastasis and lymphatic vessel formation [11, 12]. Endostatin combined with chemotherapy has been approved for the treatment of advanced non-small cell lung cancer [13]. Previous studies have shown that endostatin combined with radiotherapy achieved excellent cure rates in various tumors [14, 15]. Li et al. showed that endostatin can normalize tumor vasculature within a short time window to attenuate tumor cell hypoxia [16]. In this study, we determined whether recombinant human endostatin combined with radiotherapy can improve disease outcomes in an in vivo colorectal cancer mouse model.

## Methods

### Materials

Recombinant human endostatin was provided by Shandong Xiansheng Maidejin Biological Pharmaceutical Co., Ltd. (Shandong, China), and the colorectal cell line HCT-116 was donated by the Sixth Affiliated Hospital, Sun Yat-sen University (Guangzhou, China). The local ethics committee of Sun Yat-sen University ruled that no formal ethics approval was required in this particular case. All laboratory chemicals used herein were of molecular biology grade.

### Cell cultures

HCT-116 cells were cultured in 90% McCoy’s 5a medium with 10% fetal bovine serum (FBS) and 1% double antibiotics (100 U/mL penicillin and 100 μg/mL streptomycin) at 37 °C with 5% CO_2_. The cells were detached using trypsin-EDTA solution when they were approximately 80% confluent, after which some of them were subcultured, while the remaining cells were stored in liquid nitrogen.

### In vivo HCT-116 xenograft animal model

Forty BABL/c male nude mice aged 4–6 weeks and weighing 20 ± 2 g were purchased from Shanghai Slac Laboratory Animal Co. Ltd. (Animal Quarantine Conformity Certificate Number: 3,100,159,661, Shanghai, China) and housed under SPF conditions. All animal studies were approved by the Ethics Committee of Qingdao Central Hospital, the Second Affiliated Hospital of Qingdao University Medical College, and were performed according to the local principles of laboratory animal care. HCT-116 tumor cells in the logarithmic phase of growth (1 × 10^7^) were resuspended in 0.2 mL of serum-free medium and then injected into the upper back of each nude mouse. The volume of each implanted tumor was measured every 2 days and was calculated as V = L x W^2^/2, where L is the longer axis of the tumor, and W is the shorter axis of the tumor. Treatment was initiated when the tumors reached an approximate volume of 308 ± 56 mm^3^. The mice were randomized into the following 4 groups (each group comprised ten mice): a control group, which received peritumoral subcutaneous injections of 0.2 mL of normal saline every day for 10 days; an endostatin group, which received peritumoral subcutaneous injections of 0.2 mL of endostatin (20 mg/kg) every day for 10 days; a radiotherapy group, which received a single 6-Gy dose of external irradiation (6-MV X ray) on day 5 post-inoculation and a peritumoral subcutaneous injection of 0.2 mLl of normal saline every day for 10 days; and a combination therapy group (radiotherapy with endostatin treatment), in which endostatin was administered as in the endostatin group, and radiotherapy was administered as in the radiotherapy group. The size of the implanted tumor and the weights of the mice were monitored during this period. On day sixteen post-inoculation, all the mice were killed, and the tumors were collected. One half of each tumor was fixed in 4% neutral formalin for use in subsequent immunohistochemistry experiments, and the other half was stored at −80 °C until needed for subsequent western blotting experiments.

### Immunohistostaining

Tumor tissue sections were fixed in formalin for 24 h and then embedded in paraffin before being cut into 4-μm thick sections and stained with hematoxylin & eosin (HE). A fully automatized immunohistochemistry assay was performed on a Roche Ventana BenchMark XT (Roche Ventana Medical Systems, Tucson, Arizona,USA) using an Ultra View Universal DAB Detection Kit (Roche Ventana Medical Systems, Tucson, Arizona, USA). The following primary antibodies were used for the experiment: anti-vascular endothelial growth factor (VEGF) antibody (purified rabbit polyclonal antibody, diluted 1:80, WuXi AppTec, AP6290b-400, Suzhou, China), anti-CD31 antibody (purified rabbit polyclonal antibody, diluted 1:80, WuXi AppTec, AP7465B-400, Suzhou, China) and anti-hypoxia inducible factor-1α (HIF-1α) antibody (purified rabbit polyclonal antibody, diluted 1:80, WuXi AppTec, AP7759C-400, Suzhou, China). The tissue sections were subsequently observed under a light microscope by two experienced pathologists.

### Western blotting

The tumor tissues that had been stored at −80 °C were weighed, after which total protein was extracted from the tissues on ice using RIPA lysis buffer and a PMSF enzyme inhibitor (RIPA:PMSF = 100:1). Protein concentrations were measured using a BCA Protein Assay Kit (Beyotime Biotechnology, Shanghai, China), after which 50 μg of extracted protein and the appropriate markers were loaded onto a SDS-PAGE gel (containing a 12% separating gel and a 5% stacking gel) and then separated electrophoretically. The protein was subsequently transferred onto a PVDF membrane (Roche Applied Science, Shanghai, China), which was blocked for 1 h using 5% defatted milk diluted in Tris buffered saline Tween (TBST) before being incubated with a primary antibody to VEGF (purified rabbit polyclonal antibody, diluted 1:200, WuXi AppTec, AP6290b-400, Suzhou, China) overnight at 4 °C. The membranes were then incubated with a goat anti-rabbit IgG (H + L) HRP-conjugated secondary antibody (1:3000, LK2001, Sungene Biotech, Tianjin, China) for 1 h at room temperature. After being washed, the membranes were visualized for 1–2 min by ECL luminescence (HB-ECL-025, Hanbio, Shanghai, China), after which the protein signals in the X-ray film were quantified by scanning densitometry and analyzed by ImageJ software. The membrane was subsequently stripped with stripping buffer and then re-blocked to repeat the above steps for GAPDH, which served as an internal control (mouse monoclonal antibody, 1:1000, LK9002, Sungene Biotech, Tianjin, China). The gray value of the experimental band was compared with that of the internal reference band.

### Calculations

The tumor inhibition rate was calculated as follows: tumor inhibition rate (%) = (average volume of the control group − average volume of the experimental group)/ average volume of the control group × 100%. Brown-yellow staining indicative of positive CD31 expression was noted in the membrane and cytoplasm of vascular endothelial cells. Microvessel density (MVD) was determined by the Weidner method. First, the entire section was viewed at low power (× 100), after which the numbers of blood vessels in 5 randomly selected visual fields were counted under high power (× 200). The average number of vessels/ field was considered the MVD of the specimen. The presence of yellow or brown granules in the cytoplasm was indicative of VEGF expression. Five different visual fields were randomly selected and then viewed with the high-power microscope (× 400), and the number of positive cells and the total number of cells in each visual field were counted. The rate of positive VEGF expression for each field was calculated as follows: expression rate = number of positive cells/ total number of cells × 100%. The rate of positive VEGF expression for the sample was the mean of the 5 values mentioned above. The presence of brown-yellow granules in the nucleus was indicative of the presence of HIF-1α, whose rate of positive expression was determined in the same manner as the rate of positive VEGF expression.

### Statistical analysis

All analyses were performed with SPSS 22.0 statistical software (Softonic, San Francisco, CA, USA). The data were expressed as means ± SEMs. Comparisons among the groups were performed using Student’s t-test. A *p*-value <0.05 was considered statistically significant, and a *p*-value <0.01 was considered very significant.

## Results

### Endostatin combined with radiotherapy significantly inhibited tumor growth

**Fig. 1:**
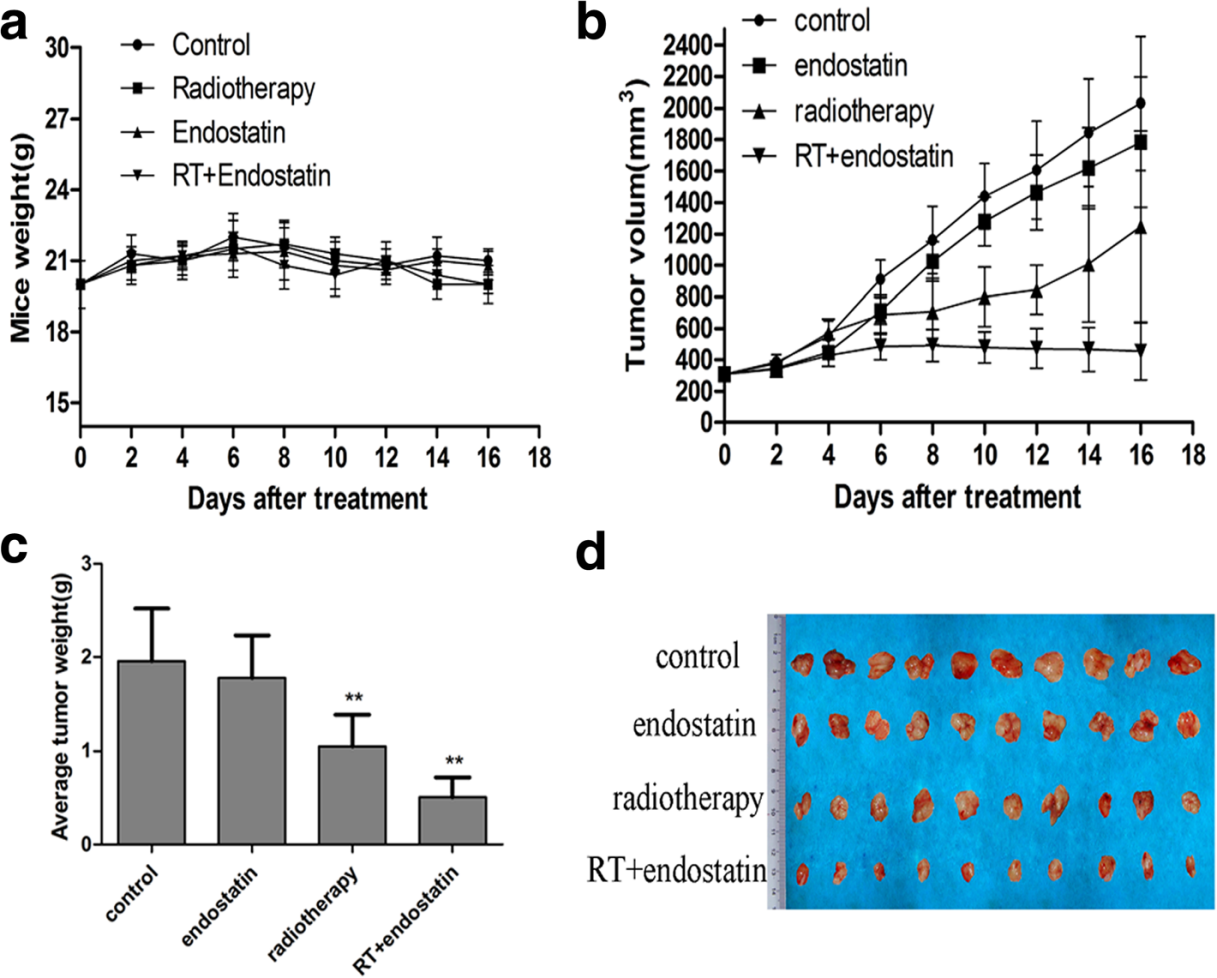
Endostatin combined with radiotherapy significantly inhibited tumor growth. **a** Changes in the average body weights of nude mice in each group during treatment (*P* > 0.05). **b** Colorectal cancer HCT-116 cell xenograft growth curves. **c** Average weights of the tumors in each group (*n* = 8 per group, **P < 0.01), compared with that in the control group. **d** Images of tumor tissue specimens dissected from mice in each group

No nude mice died during the experiment, and no differences in nude mouse diet, behavior or mental status were noted among the groups. Moreover, no significant adverse systemic reactions to the above treatments or weight changes were observed in this study. Tumor growth curves were constructed according to the average tumor volume in each group (Fig. 1). Tumor volumes tended to decline in the endostatin or radiotherapy with endostatin treatment groups compared with that in control and radiotherapy groups during the period before radiotherapy administration and after endostatin treatment; however, there was no significant difference in tumor volume among the groups (*n* = 8 per group, *P* > 0.05). Tumor growth was significantly inhibited in the radiotherapy or radiotherapy with endostatin treatment after radiotherapy. The average tumor volumes in the above groups on the 16th day of the treatment period were as follows: control group, V = 2029.95 ± 425.59 mm^3^; endostatin group, V = 1781.75 ± 412.53 mm^3^; radiotherapy group, V = 1246.60 ± 606.83 mm^3^; and radiotherapy with endostatin treatment group, V = 453.35 ± 181.43 mm^3^. We noted a significant difference in tumor volume between the radiotherapy with endostatin treatment group and the other three groups (*n* = 8 per group, *P* < 0.01). In addition, the tumor volume was significantly reduced in the radiotherapy group compared with that in the control group (*n* = 8 per group, *P* < 0.05); however, there is no significant difference in tumor volume between the endostatin and control groups. The average tumor weights in each group were as follows (g): control group, 1.96 ± 0.56; endostatin group, 1.78 ± 0.45; radiotherapy group, 1.05 ± 0.34; and radiotherapy with endostatin treatment group, 0.51 ± 0.21. There was a significant difference in tumor weight between the radiotherapy with endostatin treatment group and the other three groups (*n* = 8 per group, *P* < 0.05) (Fig. 1). On the 16th day of the treatment period, the tumor inhibition rates in the endostatin, radiotherapy and radiotherapy with endostatin treatment groups were 12.31%, 38.59% and 77.67%, respectively. Taken together, these findings indicate that endostatin combined with radiotherapy significantly inhibited HCT-116 xenograft growth.

### Pathological features of the tumor specimens in each group

**Fig. 2:**
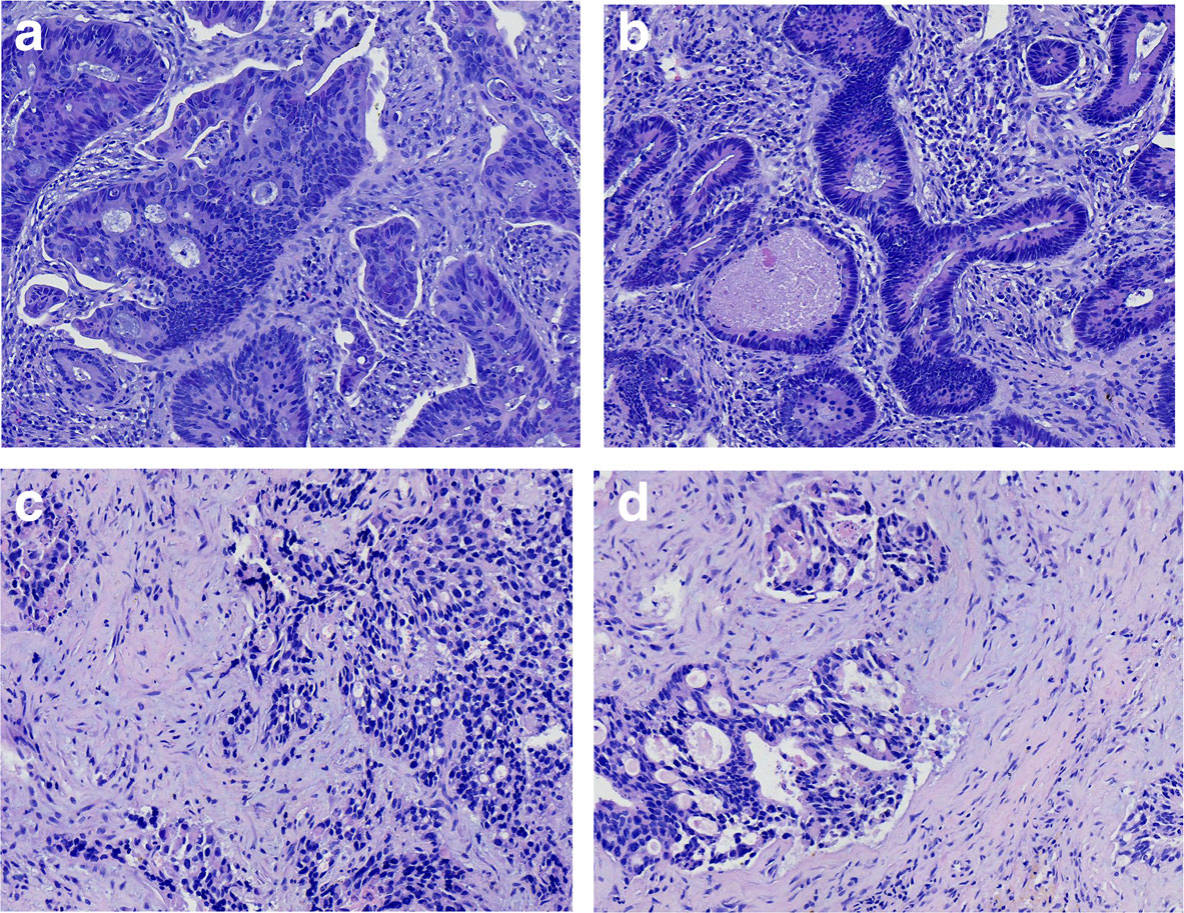
Pathological features of the tumor specimens in each group. HCT-116 cells in nude mice from the following groups (HE staining, 200×): **a**: control; **b**: endostatin; **c**: radiotherapy; **d**: radiotherapy with endostatin treatment. The tumor cells in the control group were rich and exhibited obvious atypia, while the tumor cells in other groups were replaced by fibrous tissue and displayed signs of necrosis, degeneration, and calcification

HE staining showed that many tumor cells were present in the control group and that these cells displayed obvious atypical features. However, less cells were present in the three treatment groups. Some of the cells had been replaced by fibrous tissue, while the remaining cells displayed changes consistent with necrosis, degeneration, and calcification, especially the cells in the radiotherapy with endostatin treatment group (Fig. 2).

### Effects of the indicated treatments on VEGF expression

**Fig. 3:**
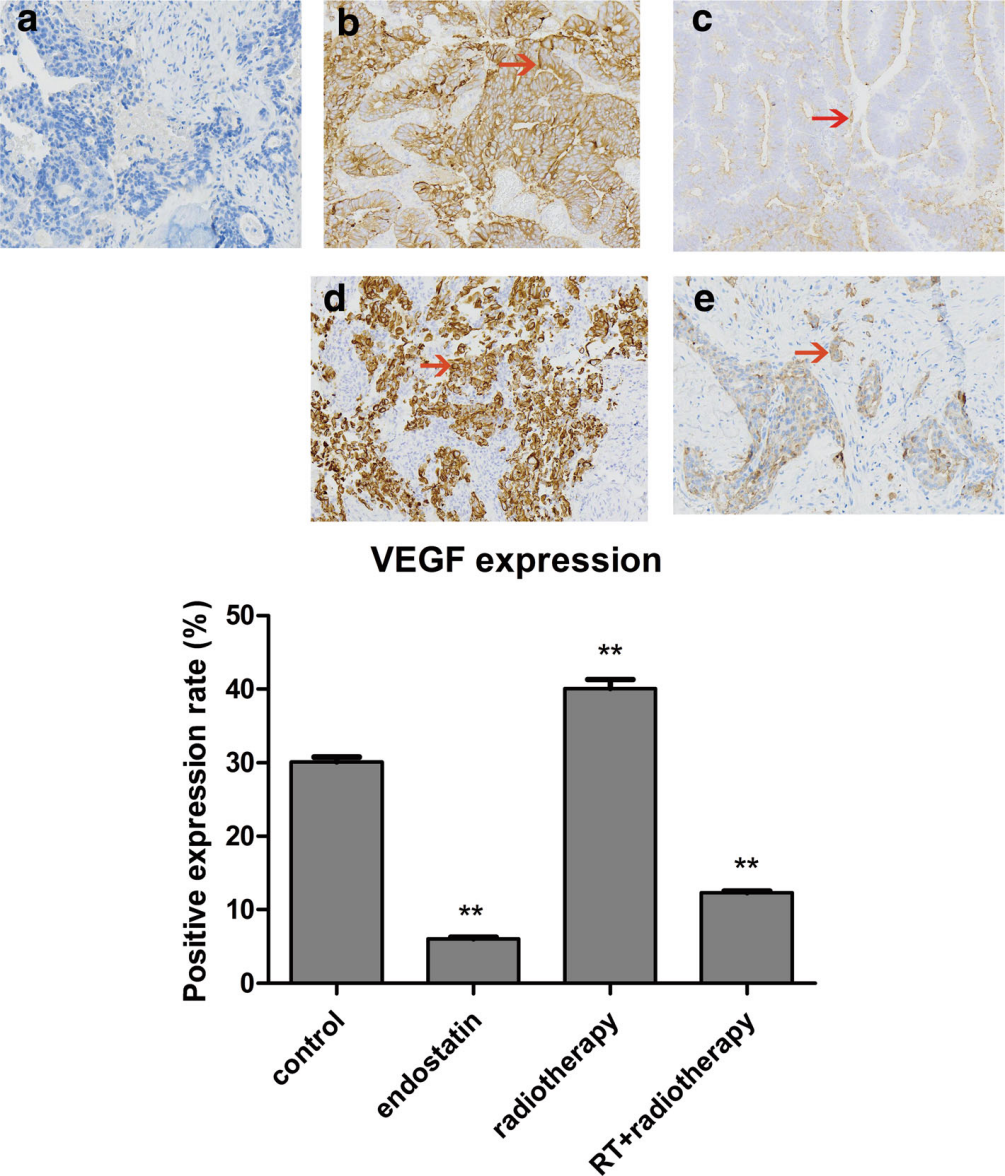
The expression and localization of VEGF by immunohistochemistry staining. **a**: control; **b**: endostatin; **c**: radiotherapy; **d**: radiotherapy with endostatin treatment. **e**: Comparison of the percentages of cells in each group that stained positive for VEGF. VEGF was localized mainly in the cytoplasm and was positively expressed in all four groups. The expression of VEGF was at a particularly high level in the radiotherapy group compared with that in other three groups. The rates of positive VEGF expression in each group were as follows: control group, 30.12% ± 0.63%; endostatin group, 6.12% ± 0.18%; radiotherapy group, 40.05% ± 1.27%; and radiotherapy with endostatin treatment group, 12.30% ± 0.25%. The intensity at which VEGF stained in the endostatin and radiotherapy with endostatin treatment groups was lower than the intensity at which it stained in the control group. In addition, the endostatin group displayed lighter VEGF staining than the other three groups (*n* = 8 per group, **P < 0.01) (200×)

**Fig. 4:**
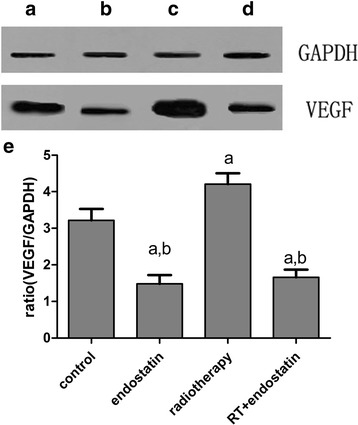
Results of western blotting. **a**: control; **b**: endostatin; **c**: radiotherapy; **d**: radiotherapy with endostatin treatment; **e**: VEGF gray value-to-GAPDH gray value ratio in each group at 16 days after treatment, ^*a*^ *P* < 0.05 versus control; ^*b*^ *P* < 0.05 versus radiotherapy (*n* = 8 per group). The western blotting results also indicated that VEGF expression was significantly increased in the radiotherapy group and significantly decreased in the endostatin and radiotherapy with endostatin treatment groups compared with the control group (*n* = 8 per group, *P* < 0.05) (200×)

The immunohistochemistry results showed that VEGF was localized mainly in the cytoplasm and that VEGF was positively expressed in all four groups (Fig. 3). Moreover, the results indicated that VEGF was expressed at a particularly high level in the radiotherapy group compared with the other three groups (*n* = 8 per group, *P* < 0.05). The rates of positive VEGF expression in each group were as follows: control group, 30.12% ± 0.63%; endostatin group, 6.12% ± 0.18%; radiotherapy group, 40.05% ± 1.27%; and radiotherapy with endostatin treatment group, 12.30% ± 0.25%. These results indicated that radiotherapy can induce VEGF expression in tumor tissues. The intensity at which VEGF stained in the endostatin and radiotherapy with endostatin treatment groups was lower than the intensity at which it stained in the control group. In addition, the endostatin group displayed lighter VEGF staining than the other three groups (*n* = 8 per group, *P* < 0.05). The western blotting results also indicated that VEGF expression was significantly increased in the radiotherapy group and significantly decreased in the endostatin and radiotherapy with endostatin treatment groups compared with the control group (*n* = 8 per group, *P* < 0.05) (Fig. 4). Taken together, these findings indicate that endostatin can inhibit VEGF expression in tumor tissues.

### Effects of each treatment on tumor MVD

**Fig. 5:**
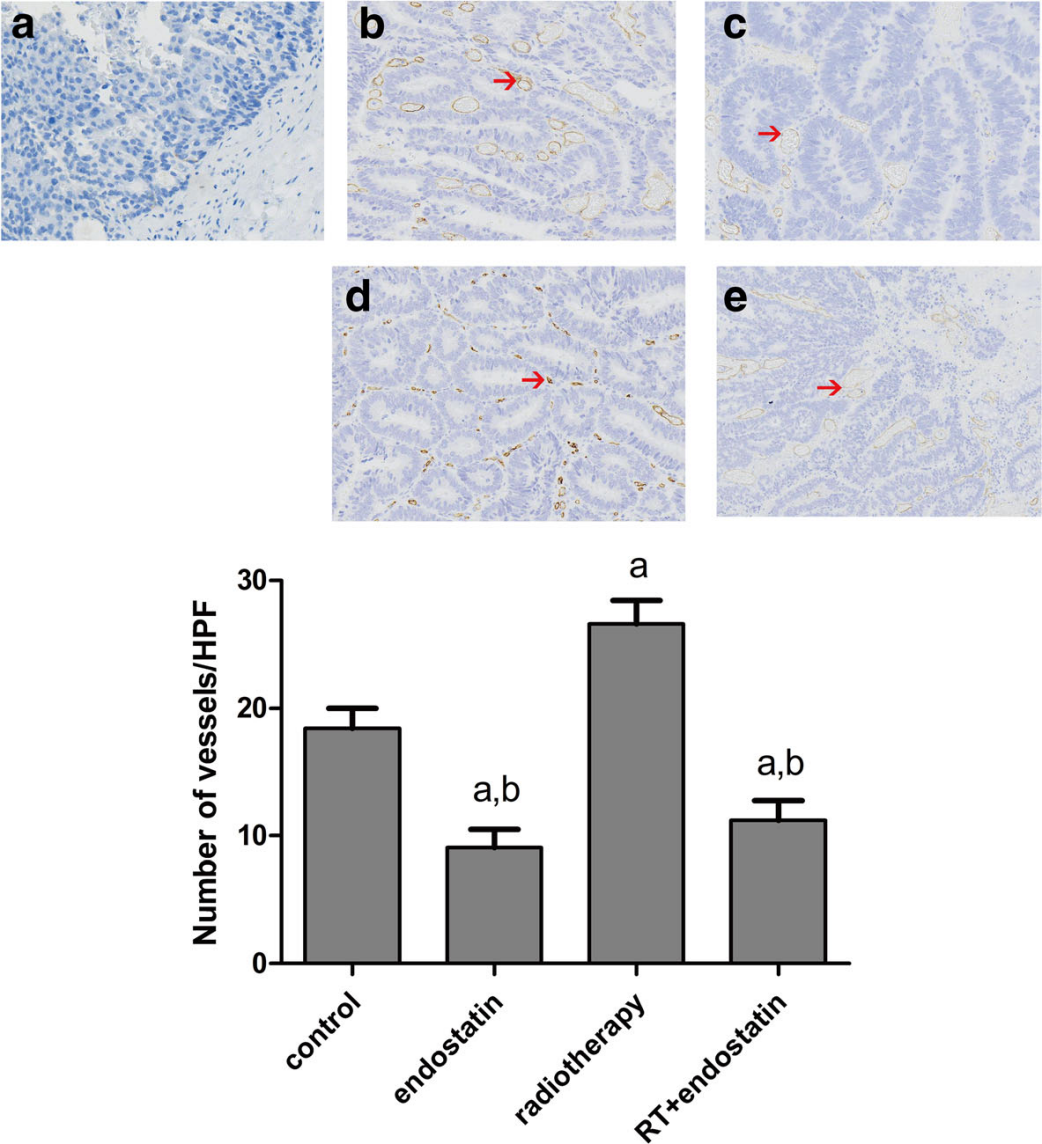
*Effects of each treatment on tumor MVD.* Expression of CD31 in each group at 16 days after treatment (**a-d**). **a**: control; **b**: endostatin; **c**: radiotherapy; **d**: radiotherapy with endostatin treatment; **e**: The MVDs of the four groups were compared by counting the numbers of CD31-positive microvessels in each group. ^*a*^ *P* < 0.05 versus control; ^*b*^ *P* < 0.05 versus radiotherapy (*n* = 8 per group). The MVD-CD31 values in the four groups were as follows: control group, 18.43 ± 1.58; endostatin group, 9.05 ± 1.46; radiotherapy group, 26.58 ± 1.86; and radiotherapy with endostatin treatment group, 11.22 ± 1.54. The MVD-CD31 value in the radiotherapy group was significantly higher than those in the other three groups (*n* = 8 per group*, P* < 0.05), and the MVD-CD31 values in the endostatin and radiotherapy with endostatin treatment groups were significantly decreased compared with that in the control group (*n* = 8 per group*, P* < 0.05) (200×)

Blood vessel proliferation in tumor tissues reflects the ability of a tumor to induce angiogenesis; thus, tumor MVD serves as an index of tumor angiogenesis. The endothelial cell marker CD31 is a new microvascular marker that can be used to identify vascular endothelial cells and to quantitatively evaluate the function of various factors that participate in angiogenesis. Additional information regarding CD31 expression is shown in Fig. 5. The MVD-CD31 values in the four groups were as follows: control group, 18.43 ± 1.58; endostatin group, 9.05 ± 1.46; radiotherapy group, 26.58 ± 1.86; and radiotherapy with endostatin treatment group, 11.22 ± 1.54. The MVD-CD31 value in the radiotherapy group was significantly higher than those in the other three groups (*n* = 8 per group*, P* < 0.05), and the MVD-CD31 values in the endostatin and radiotherapy with endostatin treatment groups were significantly decreased compared with that in the control group (*n* = 8 per group*, P* < 0.05) (Fig. 5). Taken together, these data showed that radiotherapy can induce tumor angiogenesis and that endostatin may be able to inhibit radiotherapy induced tumor angiogenesis.

### HIF-1α expression in each treatment group

**Fig. 6:**
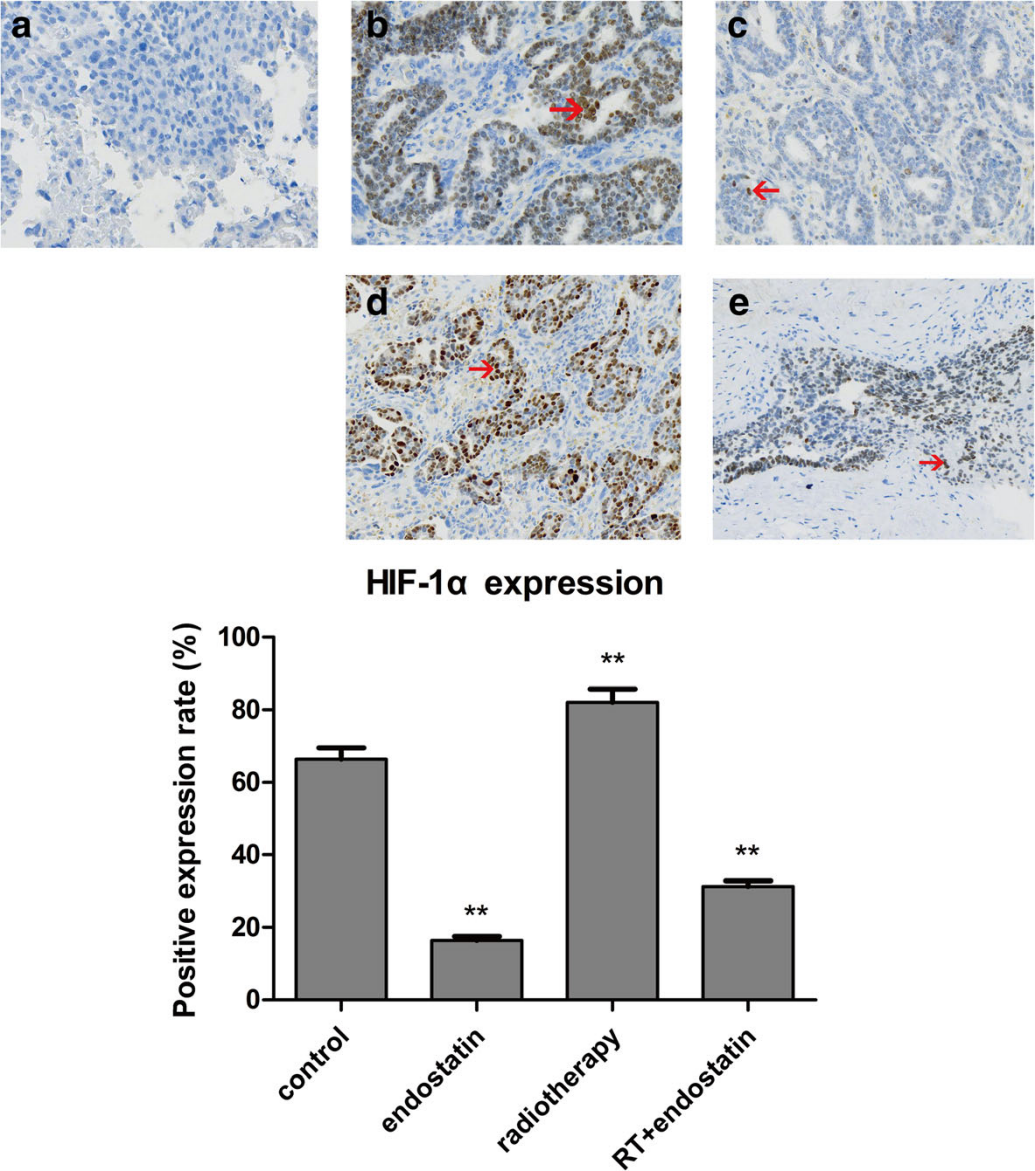
HIF-1α expression in each treatment group. **a**: control; **b**: endostatin; **c**: radiotherapy; **d**: radiotherapy with endostatin treatment; **e**: Comparison of the percentages of cells that stained positive for HIF-1α in the four groups. The rates of positive HIF-1α expression in each group were as follows: control group, 66.34% ± 3.23%; endostatin group, 16.43% ± 1.14%; radiotherapy group, 82.14% ± 3.65%; and radiotherapy with endostatin treatment group, 31.35% ± 1.51%. The rate of positive HIF-1α expression in the radiotherapy group was significantly higher than those in the other three groups (*n* = 8 per group*,P* < 0.05), while the rates of positive HIF-1α expression in the endostatin and radiotherapy with endostatin treatment groups were significantly decreased compared with that in the control group (*n* = 8 per group*,P* < 0.05) (200×)

Hypoxia is an initial inducer of malignant transformation and tumor metastasis, and HIF-1α plays an important role in regulating tumor angiogenesis. HIF-1α was strongly expressed in the control and radiotherapy groups but was expressed less strongly in the endostatin and radiotherapy with endostatin treatment groups. The rates of positive HIF-1α expression in each group were as follows: control group, 66.34% ± 3.23%; endostatin group, 16.43% ± 1.14%; radiotherapy group, 82.14% ± 3.65%; and radiotherapy with endostatin treatment group, 31.35% ± 1.51%. The rate of positive HIF-1α expression in the radiotherapy group was significantly higher than those in the other three groups (*P* < 0.05), while the rates of positive HIF-1α expression in the endostatin and radiotherapy with endostatin treatment groups were significantly decreased compared with that in the control group (*P* < 0.05) (Fig. 6). Taken together, these results indicated that endostatin may attenuate tumor hypoxia and increase the sensitivity of tumors to radiotherapy.

## Discussion

Neoadjuvant radiotherapy has been used for the treatment of rectal cancer with increasing frequency in recent years. Several studies have shown that neoadjuvant radiotherapy can lower tumor stages, improve radical resection and sphincter preservation rates and decrease local recurrence rates [17–20]. However, radiotherapy may delay surgery for patients with poor radiotherapy sensitivity. Therefore, researchers and clinicians must devise a means of improving the sensitivity of rectal cancer to radiotherapy. In our study, we found that the tumor inhibition rate in the combination therapy group was significantly higher than those in the other three groups and that no mice experienced significant increases in side effects while receiving recombinant human endostatin combined with radiotherapy as a treatment for colorectal cancer HCT-116 cell xenografts. Therefore, we determined that endostatin combined with radiotherapy is an effective method for increasing the sensitivity of cancer cells to radiotherapy.

Angiogenesis is essential for tumor growth and metastasis. Thus, clinicians and researchers must measure tumor MVD, which serves as an indicator of tumor angiogenesis [21]. VEGF plays an important role in tumor angiogenesis and is also the most important promoter of angiogenesis [22]. Hypoxia is the initial inducer of malignant transformation and tumor metastasis, and HIF-1α plays an important role in regulating tumor angiogenesis [23]. Therefore, in this study, we observed the changes in the levels of several indices of hypoxia and angiogenesis during the course of anti-tumor therapy. We found that treatment with radiotherapy induced significant increases in CD31, VEGF, HIF-1α and MVD expression compared with treatment with saline. However, treatment with endostatin and combination therapy groups induced significant decreases in CD31, VEGF, HIF-1α and MVD expression compared with treatment with saline. VEGF expression levels in the radiotherapy group were significantly increased compared with those in the control group. However, VEGF expression levels were significantly decreased in the endostatin and combination therapy groups compared with the control group. Therefore, we concluded that endostatin can inhibit radiotherapy -induced tumor angiogenesis.

Recombinant human endostatin (Endostar) is an antiangiogenesis drug that was developed independently in China. Endostar inhibits tumor angiogenesis by inhibiting the migration of endothelial cells, which form blood vessels. Endostatin can enhance tumor radiosensitivity when administered in combination with radiotherapy [14], perhaps because it can normalize tumor vasculature and the tumor microenvironment [24–26]. Winkler et al. [27] found that VEGFR2 inhibition creates a “normalization window”, i.e., a period during which radiotherapy can yield the best outcome. This window is characterized by an increase in tumor oxygenation, a change that is known to enhance tumor radiation responsiveness. Recombinant human endostatin can normalize tumor vessels for a short time period [28, 29], during which the tumor receives a normal supply of oxygen and thus becomes more sensitive to radiation. In the present study, the time window occurred mainly between days 5 and 7 after the initiation of endostatin therapy [30–34]. Therefore, radiotherapy was administered on the fifth day after the initiation of endostatin therapy in our experiment.

Wen et al. [30] concluded that endostatin combined with radiotherapy can enhance the radiosensitivity of human nasopharyngeal carcinoma and human lung adenocarcinoma xenografts, possibly by increasing endothelial cell and tumor cell apoptosis, attenuating tumor cell hypoxia, and modulating proangiogenic factor expression. Jiang et al. [31] showed that radiotherapy combined with weekly endostatin can significantly inhibit tumor growth and induce early tumor regression, phenomena that may be related to the attenuation of tumor cell hypoxia and the inhibition of radiation-induced tumor angiogenesis. Endostatin can inhibit RT-induced increases in the expression of the angiogenic factors HIF-1α and VEGF, effects that may underlie its enhancements of tumor radiosensitivity [30, 35–38]. Endostatin combined with radiotherapy has seldom been used as a treatment for advanced rectal cancers that are already being treated with well-known antiangiogenesis drugs, such as cetuximab and bevacizumab. What effect does a combination of these drugs and chemoradiotherapy have on locally advanced rectal cancer? Kim performed an analysis of two phase II trials [39] and concluded that preoperative concurrent chemoradiotherapy plus cetuximab did not improve short- or long-term therapeutic outcomes in patients with locally advanced rectal cancer and that KRAS, BRAF and PIK3CA mutations cannot predict the pathological remission rates or disease-free survival (DFS) rates of patients with the disease. Dewdney [40] analyzed 165 patients with locally advanced rectal cancer and concluded that the addition of cetuximab did not improve CR or PFS rates; however, RR and OS rates (both secondary endpoints) were significantly improved in patients with wild-type KRAS/BRAF. Willett et al. [41] conducted a multicenter phase II clinical trial, the results of which showed that bevacizumab combined with preoperative concurrent chemoradiotherapy significantly improved DFS and OS and that the acute and postoperative toxicity induced by the regimen was also acceptable. However, oncologists should be aware that bevacizumab causes surgical wound healing delays, anastomotic fistulae and bleeding. Cetuximab and bevacizumab have been shown to have some therapeutic effects on locally advanced rectal cancer; however, those drugs are so expensive that many patients in China cannot afford them. Endostatin is much cheaper than the above drugs; thus, we conducted this study to determine whether endostatin combined with radiotherapy can have positive effects in an in vivo colorectal cancer mouse model. We observed that endostatin combined with radiotherapy can significantly inhibit HCT-116 cell xenograft growth, possibly by inhibiting angiogenesis and attenuating tumor cell hypoxia. Our study serves a theoretical basis for the application of the combination therapy regimen discussed herein in clinical practice.

## Conclusions

Endostatin combined with radiotherapy can significantly inhibit HCT-116 cell xenograft growth, possibly by inhibiting angiogenesis and attenuating tumor cell hypoxia.

## Abbreviations

DFS: Disease-free survival
Endostar: Recombinant human endostatin
HIF-1α: Hypoxia inducible factor-1α
MVD: Microvascular density
VEGF: Vascular endothelial growth factor
